# Current-direction/amplitude-dependent single channel gating kinetics of mouse pannexin 1 channel: a new concept for gating kinetics

**DOI:** 10.1038/s41598-017-10921-x

**Published:** 2017-09-05

**Authors:** Takeshi Nomura, Akiyuki Taruno, Makoto Shiraishi, Takashi Nakahari, Toshio Inui, Masahiro Sokabe, Douglas C. Eaton, Yoshinori Marunaka

**Affiliations:** 1 Department of Molecular Cell Physiology, Kyoto Prefectural University of Medicine Graduate School of Medical Science, Kyoto, 602-8566 Japan; 2 Department of Bio-Ionomics, Kyoto Prefectural University of Medicine Graduate School of Medical Science, Kyoto, 602-8566 Japan; 3 Department of Physical Therapy, Faculty of Rehabilitation, Kyushu Nutrition Welfare University, Kitakyushu, 800-0298 Japan; 4 Japan Institute for Food Education and Health, St. Agnes’ University, Kyoto, 602-8013 Japan; 5 Saisei Mirai Clinics, Moriguchi, 570-0012 Japan; 6 Mechanobiology Laboratory, Nagoya University Graduate School of Medicine, Nagoya, 466-8550 Japan; 7 Center for Cell & Molecular Signaling, Department of Physiology, Emory University School of Medicine, Atlanta, Georgia 30322 USA

## Abstract

The detailed single-channel gating kinetics of mouse pannexin 1 (mPanx1) remains unknown, although mPanx1 is reported to be a voltage-activated anion-selective channel. We investigated characteristics of single-channel conductances and opening and closing rates of mPanx1 using patch-clamp techniques. The unitary current of mPanx1 shows outward rectification with single-channel conductances of ~20 pS for inward currents and ~80 pS for outward currents. The channel open time for outward currents (Cl^−^ influx) increases linearly as the amplitude of single channel currents increases, while the open time for inward currents (Cl^−^ efflux) is constant irrespective of changes in the current amplitude, as if the direction and amplitude of the unitary current regulates the open time. This is supported by further observations that replacement of extracellular Cl^−^ with gluconate^−^ diminishes the inward tail current (Cl^−^ efflux) at a membrane potential of −100 mV due to the lowered outward current (gluconate^−^ influx) at membrane potential of 100 mV. These results suggest that the direction and rate of charge-carrier movement regulate the open time of mPanx1, and that the previously reported voltage-dependence of Panx1 channel gating is not directly mediated by the membrane potential but rather by the direction and amplitude of currents through the channel.

## Introduction

**Figure 1:**
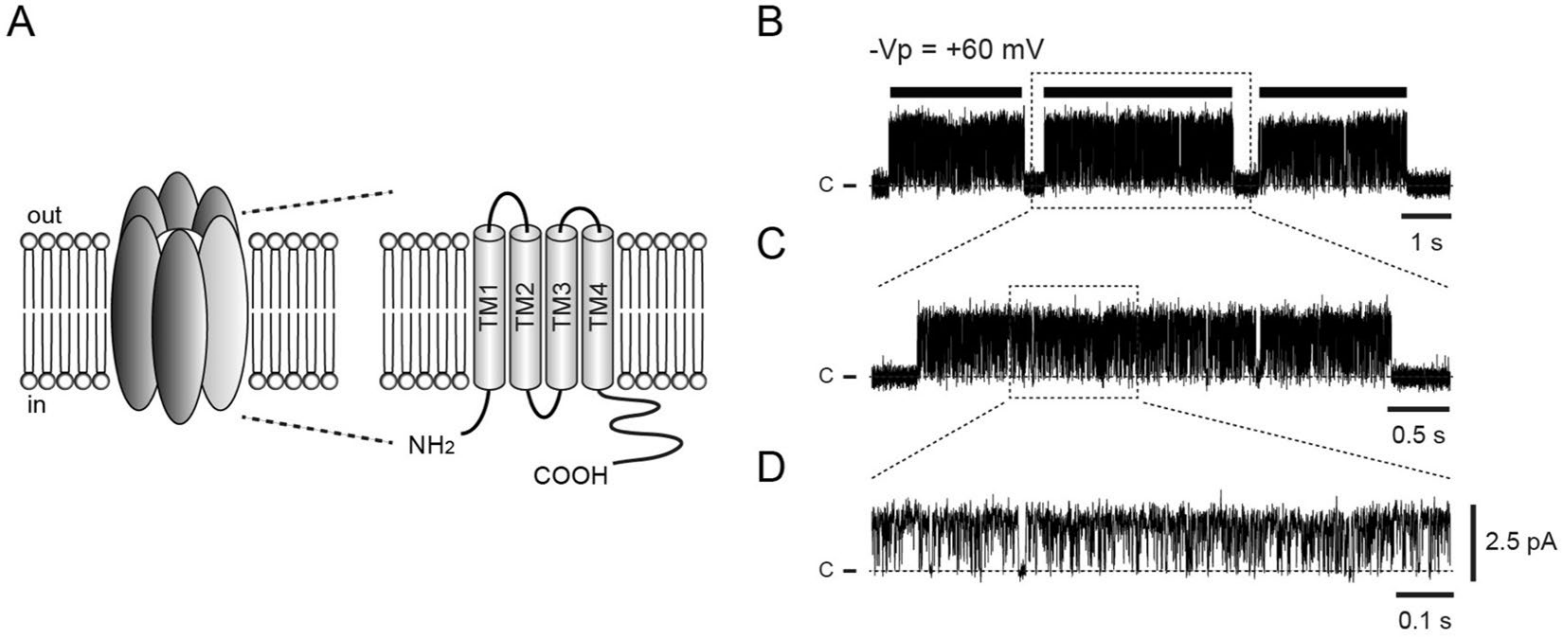
Cartoon of mPanx1 and singe-channel current traces. (**A**) A mPanx1 channel is composed of six subunits (left) with the membrane topology of a Panx1 monomer shown on right. (**B**) Representative single-channel current traces of mPanx1 (upper trace) recorded at −60 mV pipette potential obtained from cell-attached patches (−Vp = +60 mV; the patch membrane was clamped at +60 mV ‘positive’ deviated from the resting membrane potential). A burst is indicated by horizontal bar in (**B**). (**C**) Middle trace (**C**) is expanded view of upper trace (**B**). (**D**) Lower trace (**D**) is magnification middle trace (**C**). “c” and dashed lines represent the closed levels of single-channel currents. Within a burst, single mPanx1 channels switch rapidly between closed and open states.

Pannexins (Panx) have been identified as channel proteins^1, 2^ that have homology to invertebrate gap-junction forming proteins, innexins^1, 2^, but have no homology to the vertebrate connexin gap-junction proteins^3, 4^. The Panx family is composed of three members, Panx1, Panx2 and Panx3 proteins^1^. Panx1 is ubiquitously expressed in various mammalian tissues^5^. Unlike connexins, Panx1 channels do not form gap-junctions in the plasma membrane^6–9^. Nevertheless, similar to connexins, the Panx1 channel is a hexamer^10^ of Panx1 monomers, each of which contains four transmembrane domains, two extracellular loops, and cytoplasmic N- and C-termini^7, 10, 11^ (Fig. 1A). Each subunit has a molecular mass of ~48 kDa^12^ and consists of 426 amino acids^8, 12^. Panx1 forms a large pore channel that conducts small molecules up to ~1 kDa in molecular weight^13, 14^, and has been proposed to act as a conduit for ATP release^7, 15–19^.

The Panx1 channel is activated by membrane depolarization^2, 8^, mechanical stretch^15^ and extracellular ATP binding to purinergic receptors including the P2X_7_ receptor which interact with Panx1^20, 21^. Furthermore, recent studies have proposed that activation of Panx1 channels by caspase 3/7 cleavage of its C terminus during apoptosis induces ATP release as a ‘find-me’ signal to recruit macrophages to apoptotic cells^22–25^. Panx1 currents exhibit outward rectification^2, 8^ and anion selectivity^26^. Replacement of extracellular Cl^−^ with other anions induces a shift of the whole-cell current reversal potential and alters whole-cell current amplitudes: the anion selectivity sequence of mPanx1 channel has been reported to be NO_3_^−^ > I^−^ > Br^−^ > Cl^−^ > F^−^ >> aspartate^−^ ≈ glutamate^−^ ≈ gluconate^−^^26^. Channel proteins such as acid-sensing ion channels (ASICs)^27^ and epithelial Na^+^ channel (ENaC)^28^ have binding sites for Cl^−^, and activity of these channels is modulated by Cl^−^ binding^27, 28^, which plays essential roles in regulation of cell function^29–39^.

The gating mechanism of Panx1 channels is of great interest. The whole-cell currents of Panx1 are augmented by membrane depolarization^8, 26, 40^ and the open probability of single mPanx1 channel also increases in a voltage-dependent manner^40^, implying that Panx1 gating is regulated by membrane potential. Because of its voltage-dependent gating, one may expect that the transmembrane domains of mPanx1 contain charged amino acids as a voltage sensor. However, in fact, its transmembrane domains lack such a canonical voltage sensor^41^. Thus, the mechanism of the voltage-dependent gating of Panx1 remains unknown.

In this study, we examined the conductance and gating properties of single mPanx1 channels expressed in HEK293 cells using the patch-clamp technique. Our observations indicate that the open time of the channel for outward currents increases linearly as the amplitude of single channel currents increases, while the open time of the channel for inward currents is constant irrespective of changes in the amplitude of single channel current. These results suggest that the direction and amplitude of currents regulates the open time (closing rate) of mPanx1 channels, and that the previously reported voltage-dependence of Panx1 channel gating is not directly due to the membrane potential but rather due to the direction and amplitude of the single channel current driven by membrane potential. This concept is supported by our observation of reduced inward tail currents (Cl^−^ efflux) caused by diminution of outward currents after replacement of extracellular Cl^−^ with gluconate^−^ (to which Panx1 channels are less permeable than Cl^−^). The present study proposes a novel concept that the direction and magnitude of ion flux through the channel pore regulates the gating. These data have been partly reported in abstract form^42^.

## Materials and Methods

### Cell culture and transfection

Human embryonic kidney (HEK) 293 cells and mouse neuroblastoma Neuro2a cells were grown in Dulbecco’s Modified Eagle’s Medium (DMEM) (Sigma-Aldrich, St. Louis, MO, USA) and Minimum Essential Medium (MEM) (Gibco), respectively, containing 10% fetal bovine serum (FBS), 100 µg/ml streptomycin and 100 units/ml penicillin (Wako Pure Chemical, Osaka, Japan) in a humidified atmosphere at 37 °C with 5% CO_2_. For expression of recombinant mPanx1 and mPanx1Δ371, HEK293 and Neuro2a cells were transiently transfected with a *Panx1*-containing expression plasmid, pIRES2-EGFP^40^, using Lipofectamine 2000 or Lipofectamine 3000 transfection reagents (Invitrogen, Carlsbad, CA, USA) following the manufacturer’s specifications. Electrophysiological experiments were performed 24~48 h after transfection.

### Single-channel current recordings

All experiments were performed in the cell-attached configuration of the patch-clamp technique at room temperature (23~25 °C). The pipette solutions contained (in mM) 140 NaCl (140 NaF, 140 NaNO_3_, 140 NaBr, 140 NaSCN or 140 Na-gluconate), 2 KCl, 1 CaCl_2_, 1 MgCl_2_ and 10 HEPES (pH 7.4 adjusted with NaOH). The bath solution contained 140 NaCl, 2 KCl, 1 CaCl_2_, 1 MgCl_2_ and 10 HEPES (pH 7.4 adjusted with NaOH). Borosilicate glass pipettes (Drummond Scientific Co., Broomall, PA, USA) were pulled using a pipette puller (PP-830, Narishige, Tokyo, Japan) and polished with a microforge (MF-9, Narishige, Tokyo, Japan) to a diameter corresponding to a pipette resistance within a 7.2~9.2 MΩ range in the NaCl pipette solution. Currents were amplified with an Axopatch 200B amplifier (Molecular Devices, Sunnyvale, CA, USA). Data were acquired at 5 kHz with a Digidata 1322A interface using pCLAMP 10 acquisition software (Molecular Devices, Sunnyvale, CA, USA), and stored in a personal computer. The single channel current traces were low-pass filtered at 1 kHz through a Bessel filter. Single-channel mPanx1 channel currents occur in bursts (Fig. 1B and C) with fast open-closed kinetics within bursts (Fig. 1D). In the present study, we determined the unitary conductance from the slope of the current-voltage relationship and the single channel gating kinetics in cell-attached patches by analyzing the single-channel currents within bursts.

### Open probability (P_o_) of a single channel

Single-channel currents were analyzed using pCLAMP 10 software (Molecular Devices, Sunnyvale, CA, USA). We determined the number of active channels per patch membrane (*N*) as reported in our previous study^43^. We considered “*N*” as the true number of active channels per a patch membrane with a probability <0.001 that the patch contained more than “*N*” channels^43^. The open probability (P_o_) was calculated from the cell-attached patch-clamp recordings in a patch membrane using the following equation.

$${{\rm{P}}}_{{\rm{o}}}=\frac{1}{N{{\rm{T}}}_{{\rm{R}}}}\sum _{i=1}^{N}i\,{T}_{oi},$$

where T_o*i*_ is the total time of a channel staying at the open state “*i”* (*i* means the number of channels simultaneously opened: e.g., “*i* =  2” means that only 2 channels simultaneously opened) during the total recording time period (T_R_) within bursts.

### Closed and open times of a single channel

To determine mean closed and open times of a single channel, we created histograms for closed and open dwell times within bursts obtained from single channel recordings where the patch membrane was considered to contain only one active channel with a probability <0.001 that the patch membrane contained more than one active channel as described in our previous study^43^. Then, we obtained time constants by performing maximal likelihood fitting with a single exponential for the closed and open dwell time histograms, respectively. The time constants determined from the closed and open dwell time histograms are respectively the mean closed and open times.

### Opening and closing rates of a single channel

The opening and closing rates were calculated from the single-channel recordings in a patch membrane using the following equations.

$${\rm{Opening}}\,{\rm{rate}}=\frac{1}{{\rm{Closed}}\,{\rm{time}}},$$

$${\rm{Closing}}\,{\rm{rate}}=\frac{1}{{\rm{Open}}\,{\rm{time}}},$$

where the opening rate is the rate of the channel leaving the closed state and the closing rate is the rate of the channel leaving the open state. The closed and open times were determined by the method described above (see the section of closed and open times of a single channel).

### Apparent voltage-dependency of channel gating

The apparent V_1/2_ and number of channel gating charges were determined by performing maximal likelihood fitting with Boltzmann equation for the open probabilities at various voltages.

### Whole cell current recordings

Whole cell currents were obtained with 3.0~4.5 MΩ borosilicate glass pipettes from Neuro2a cells transiently transfected with mPanx1Δ371. Cells were continuously perfused (~3 mL/min) at room temperature (23~25 °C) with a bath solution (pH 7.4) containing (in mM) 140 NaCl, 5 KCl, 2 CaCl_2_, 1 MgCl_2_, 10 glucose and 10 HEPES. To substitute extracellular Cl^−^ with gluconate^−^, NaCl was replaced with equimolar Na-gluconate. The intracellular solution contained 140 CsCl, 5 NaCl, 5 MgCl_2_, 5 EGTA and 10 HEPES (pH 7.4). mPanx1Δ371-expressing cells were identified by GFP fluorescence. Whole-cell currents were recorded with an Axopatch 1D amplifier and a Digidata 1321A interface using pCLAMP 8 acquisition software (Molecular Devices, Sunnyvale, CA, USA). The current data were low-pass filtered at 500 Hz with a four-pole Bessel filter and digitized at 2 kHz. Cells were held at −100 mV and ramp voltage pulses (−100 mV to +100 mV over 500 ms; +100 mV for 125 ms) were applied at 5 s intervals.

### Statistical analysis

All values are shown as mean ± SEM. The Student’s *t*-test was used for statistical analysis as appropriate. *P* value < 0.05 was considered as a statistically significant difference.

## Results

### Single-channel current and unitary conductance of mPanx1 channel

**Figure 2:**
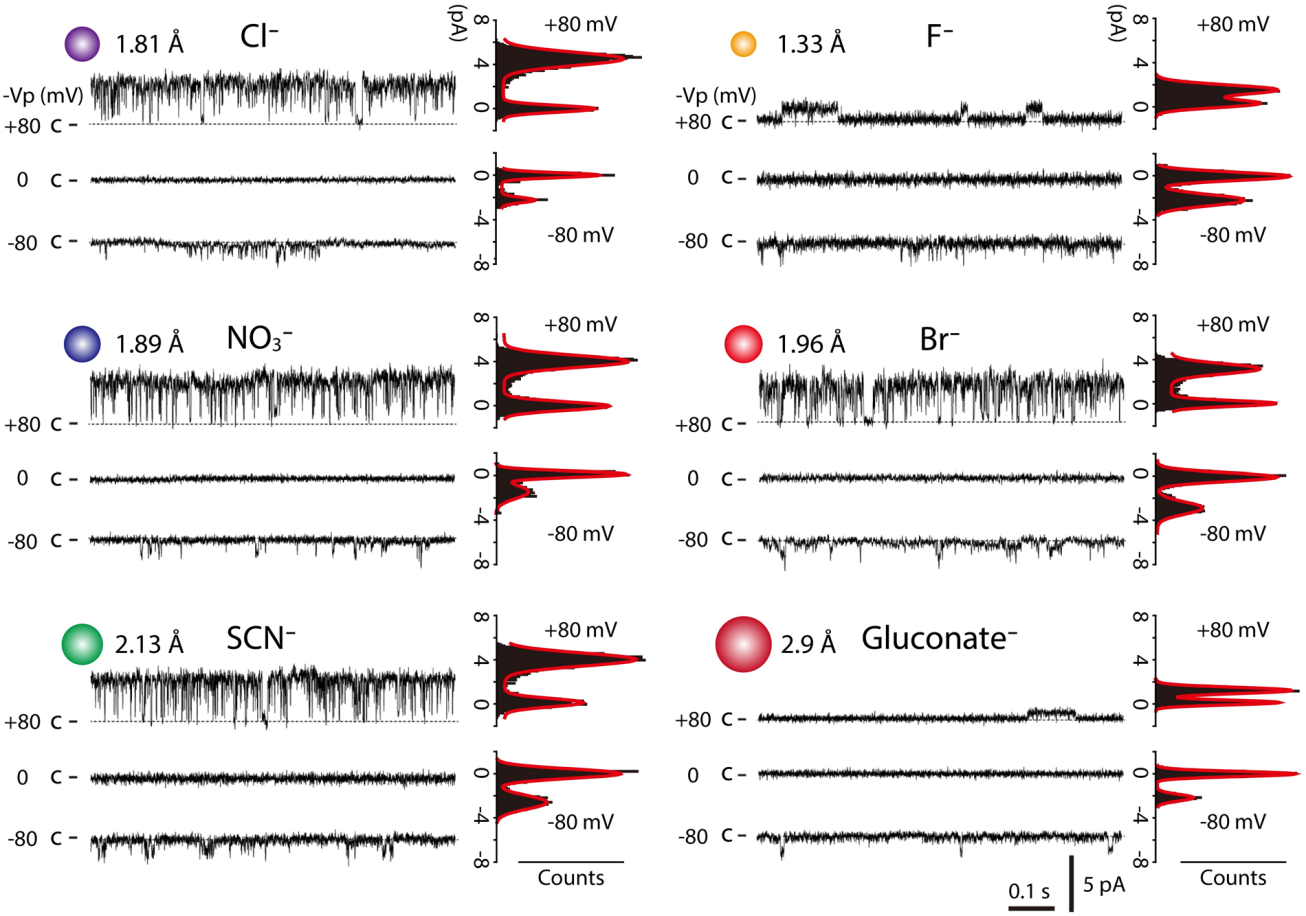
Single-channel currents of mPanx1 channel in the presence of various anions in the pipette solution obtained from cell-attached patches. Representative single-channel currents in the presence of various anions in the pipette solution recorded at −Vp ± 80 mV. Numbers shown on left of the single-channel current traces indicate applied potential to the patch pipette (−Vp). “c” and dashed lines indicate channel closed state in patches under conditions where the bath solutions contained (in mM) 140 NaCl, 2 KCl, 1 CaCl_2_, 1 MgCl_2_ and 10 HEPES, and the pipette solutions contained (in mM) 140 NaCl, 140 NaF, 140 NaNO_3_, 140 NaBr, 140 NaSCN or 140 Na-gluconate in addition to 2 KCl, 1 CaCl_2_, 1 MgCl_2_ and 10 HEPES. The ionic radius of each anion is shown^57, 58^. All-point amplitude histograms for currents at −Vp = ±80 mV are shown on the right of current traces. Amplitude histograms were fitted by two Gaussian distributions (red lines).

The single channel activity of mPanx1 was examined during application of negative and positive pipette potentials in cell-attached membrane patches in HEK293 cells. Figure 2 shows representative single-channel current traces of mPanx1 channels in the presence of various anions (Cl^−^, F^−^, NO_3_^−^, Br^−^, SCN^−^ and gluconate^−^) in the pipette solution at −Vp of ±80 mV (−Vp is the displacement of the patch potential from the resting intracellular potential, e.g. −Vp = +80 mV means that the patch membrane was clamped at +80 mV more positive than the resting membrane potential, while −Vp = −80 mV means that the patch membrane was 80 mV more negative than the resting membrane potential; −Vp of 0 mV means that the patch membrane was clamped at the resting membrane potential). The single-channel conductance (~80 pS) for outward Cl^−^ currents is consistent with previous reports^26, 40^. Such channels were not observed in mock-transfected HEK293 cells.

**Figure 3:**
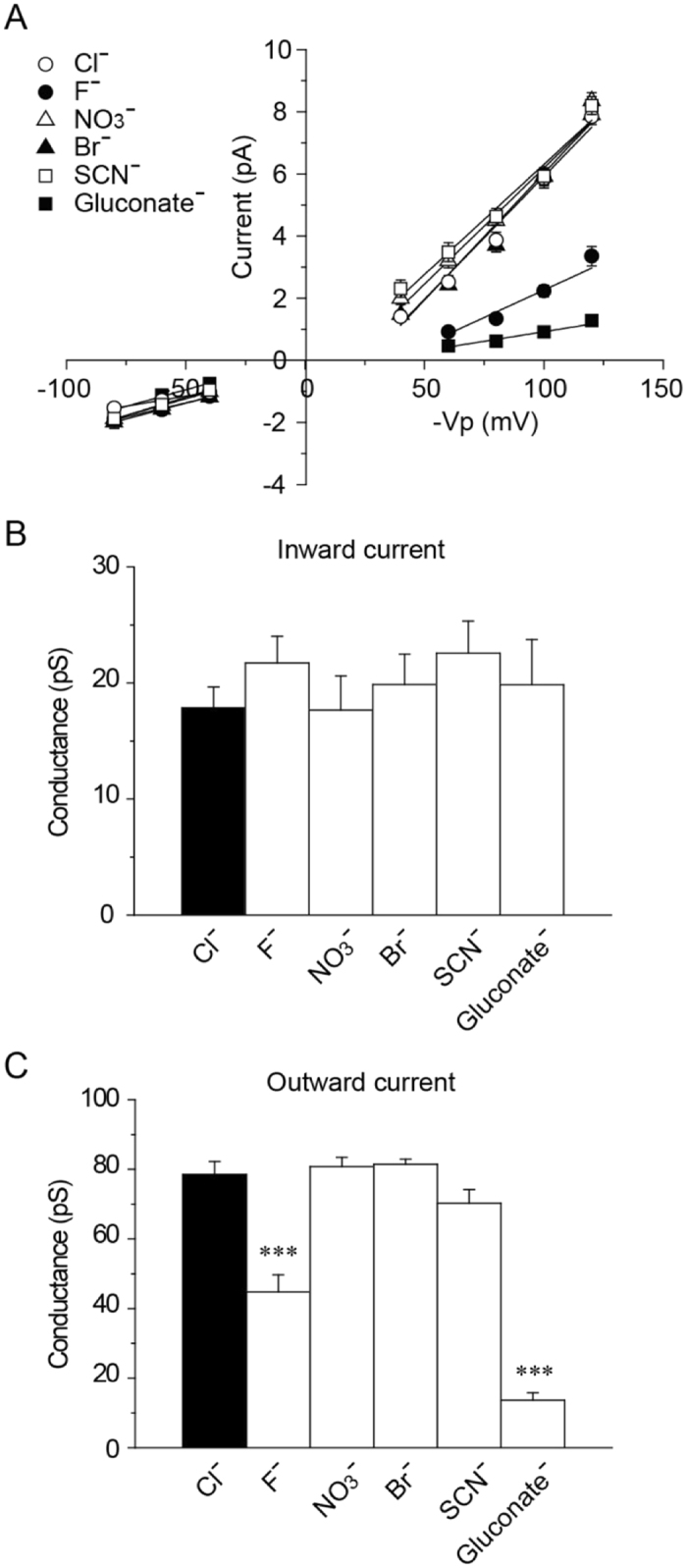
Current-voltage relation of mPanx1 channel with various anions in the pipette solution. (**A**) Current and voltage relationships of mPanx1 channel under conditions where bath solutions contained (in mM) 140 NaCl, 2 KCl, 1 CaCl_2_, 1 MgCl_2_ and 10 HEPES, and pipette solutions contained (in mM) 140 NaCl, 140 NaF, 140 NaNO_3_, 140 NaBr, 140 NaSCN or 140 Na-gluconate in addition to 2 KCl, 1 CaCl_2_, 1 MgCl_2_ and 10 HEPES. Outward rectification was seen in the presence of various anions except gluconate^−^. Unitary conductance of mPanx1 channel in inward (**B**) (n = 6~7) and outward (**C**) currents (n = 7~9) is shown for different anions. Asterisk indicates significant difference from Cl^−^ (***P < 0.001). Each value is represented as mean ± SEM.

The single mPanx1 channel current exhibited weak outward rectification for all anions (Fig. 3A)^40, 44^. The single-channel conductances for inward and outward currents were calculated from the current-voltage curves obtained at applied pipette potentials ranging from −80 to +80 mV (−Vp = −80 to +80 mV) (Fig. 3A). The single-channel conductance for inward currents was 17.8 ± 1.8 pS in Cl^−^, 21.7 ± 2.3 pS in F^−^, 17.7 ± 2.9 pS in NO_3_^−^, 19.9 ± 2.6 pS in Br^−^, 22.6 ± 2.8 pS in SCN^−^ and 19.8 ± 3.9 pS in gluconate^−^, respectively (*n* = 6~7) (Fig. 3B). No significant differences were observed between Cl^−^ and other anions. On the other hand, the unitary conductances for outward currents were 78.4 ± 3.8 pS in Cl^−^, 44.8 ± 4.9 pS in F^−^, 80.8 ± 2.7 pS in NO_3_^−^, 81.4 ± 1.5 pS in Br^−^, 70.3 ± 3.9 pS in SCN^−^ and 13.7 ± 2.1 pS in gluconate^−^, respectively (*n* = 7~9) (Fig. 3C). The single-channel conductances for outward F^−^ and gluconate^−^ currents were significantly lower than that for Cl^−^ currents. It should be noted that the inward currents were generated by Cl^−^ in the cytoplasm, while the outward currents were generated by Cl^−^, F^−^, NO_3_^−^, Br^−^, SCN^−^ or gluconate^−^ present in the pipette solution.

### Apparent voltage dependent gating of mPanx1

**Figure 4:**
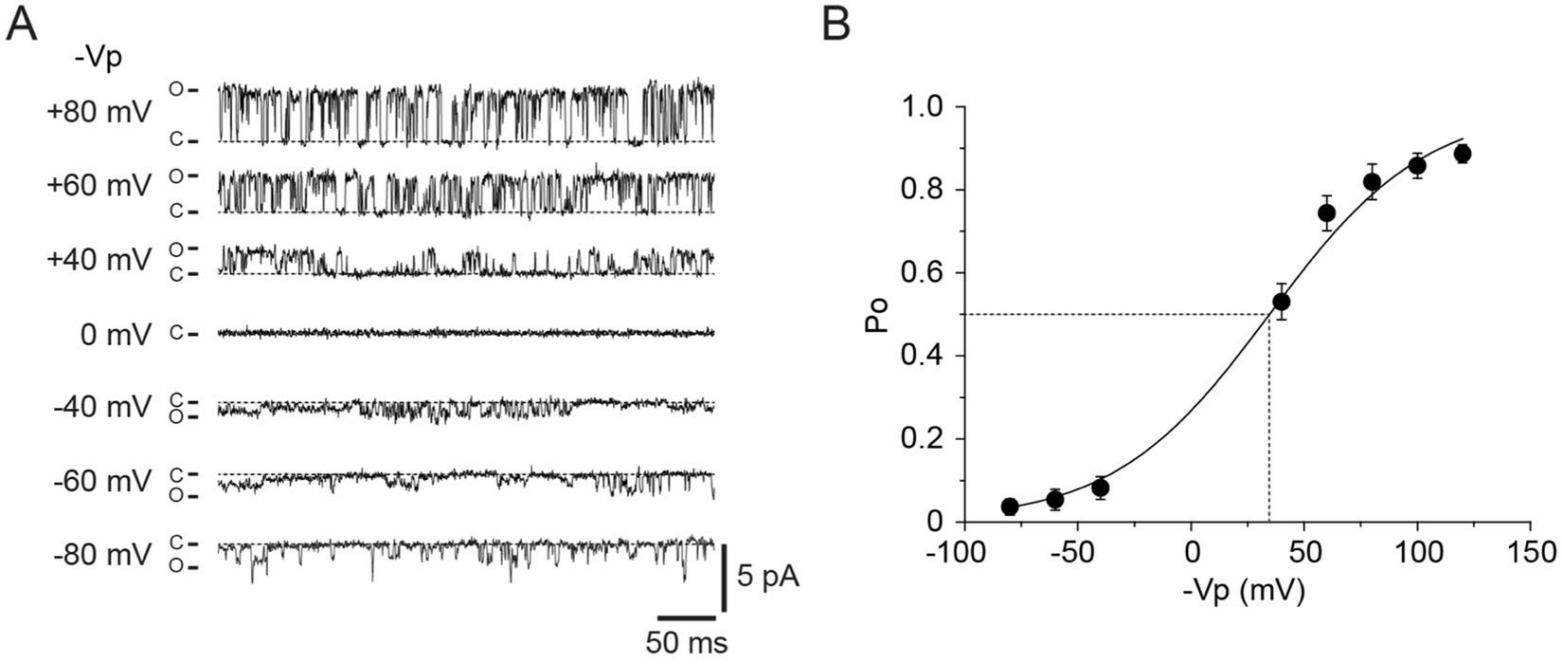
P_o_-voltage relation of mPanx1 channel obtained from cell-attached patches under a condition where the pipette and bath solutions contained (in mM) 140 NaCl, 2 KCl, 1 CaCl_2_, 1 MgCl_2_ and 10 HEPES. (**A**) Representative single-channel current traces of mPanx1 recorded at −Vp of ±40, ±60 and ±80 mV obtained from cell-attached patches. (**B**) P_o_-voltage relation for mPanx1 channel. The solid line was obtained using Boltzman fit. Dotted line represents average midpoint voltage (−Vp_1/2_) of mPanx1 channel for Cl^−^ (34.3 ± 5.8 mV). Data are shown as mean ± SEM (*n* = 8).

Representative single-channel currents of mPanx1 recorded at −Vp of ±40, ±60 and ±80 mV obtained from cell-attached patches with the pipette and bath solutions containing Cl^−^ are shown in Fig. 4A. P_o_ increased with membrane depolarization (Fig. 4B). The plots were fitted with Boltzmann equation to obtain the apparent half maximal activation voltage (V_1/2_) and the number of gating charges. The apparent V_1/2_ of the channel in the presence of Cl^−^ was at −Vp of 34.3 ± 5.8 mV (*i.e*. 34.3 ± 5.8 mV more positive potential from the resting potential; *n* = 8). The apparent number of gating charges was 0.89 ± 0.07 (*n* = 8). Thus, it appears that mPanx1 gating is dependent on membrane voltage despite a lack of canonical voltage sensors, suggesting that there are as-yet-unknown mechanisms in the regulation of mPanx1 gating.

### Current-direction/amplitude-dependency of opening and closing rates of mPanx1 channel at various voltages

**Figure 5:**
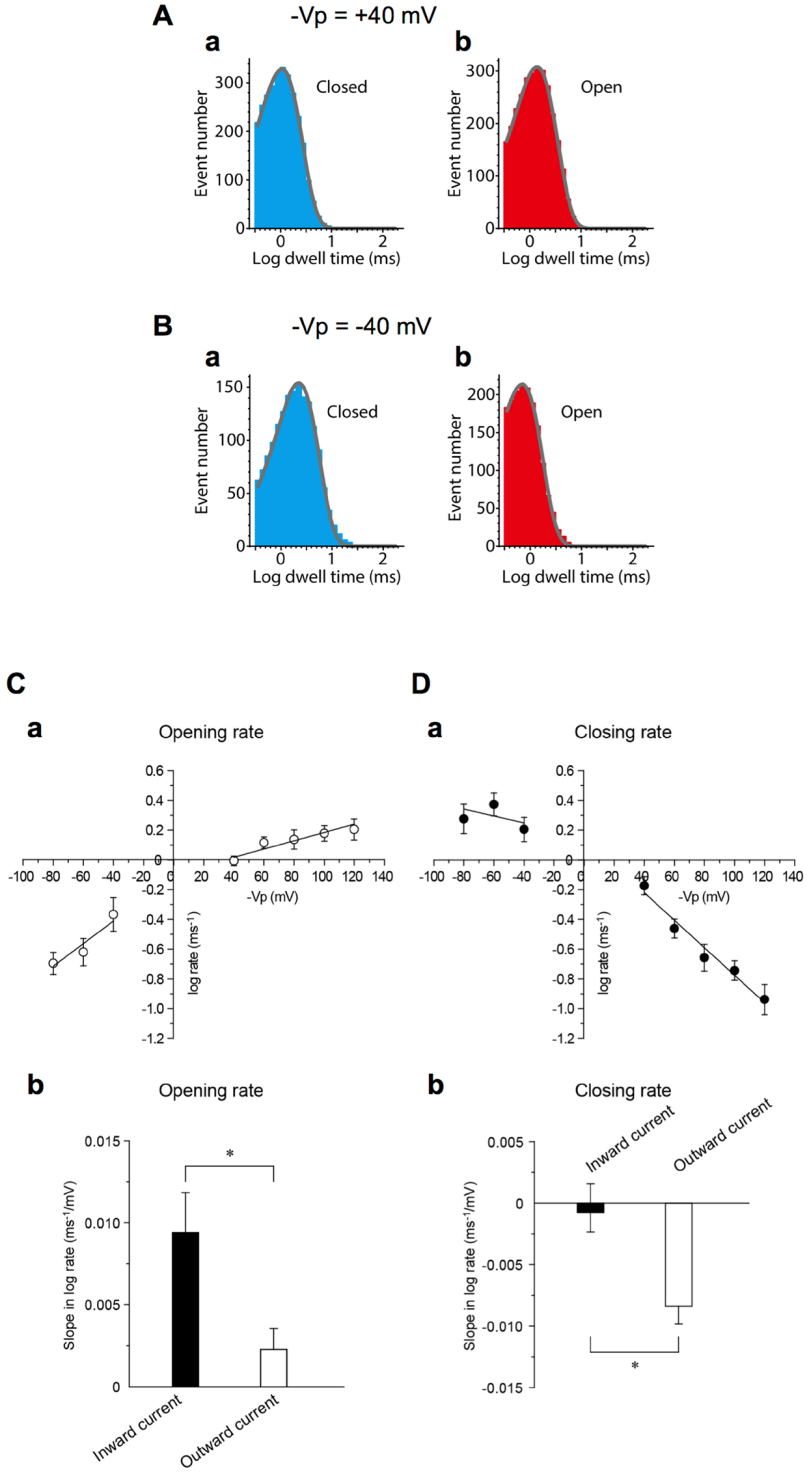
Single-channel kinetic analysis for mPanx1 channel. mPanx1 channel recordings were made with pipette and bath solutions containing (in mM) 140 NaCl, 2 KCl, 1 CaCl_2_, 1 MgCl_2_ and 10 HEPES. (**A**-a) A typical histogram of closed dwell time at −Vp = +40 mV. (A-b) A typical histogram of open dwell time at −Vp = +40 mV. (**B**-a) A typical histogram of closed time at −Vp = −40 mV. (**B**-b) A typical histogram of open dwell time at −Vp = −40 mV. The gray continuous lines in histograms are single exponential fits to the data. Opening (**C**) and closing (**D**) rates at various voltages. (**C**-a) Opening rates increase under both inward current (−Vp = −80 to −40 mV: hyperpolarized) and outward current (−Vp = +40 to +120 mV: depolarized) conditions. (**C**-b) Slope of the increase of opening rate under inward current (−Vp = −80 to −40 mV: hyperpolarized) conditions was larger than that at outward current (−Vp = +40 to +120 mV: depolarized) conditions. (**D**-a and b) In contrast, the closing rate decreased under outward current (−Vp = +40 to +120 mV: depolarized) conditions, whereas under inward current (−Vp = −80 to −40 mV: hyperpolarized) conditions it was unchanged. This indicates that single-channel kinetics of mPanx1 drastically changes at the reversal potential at which the current direction through the channel reverses. Data are shown as mean ± SEM (n = 6).

Figure 5A and B respectively show typical histograms for closed and open dwell times at −Vp = +40 mV and −Vp = −40 mV with Cl^−^ in the pipette and bath solutions. Each histogram was well fitted with a single exponential. The opening and closing rates at various voltages are shown in Fig. 5C and D. The opening rate monotonically increased within the voltage range tested (−Vp = −80 to +120 mV) (Fig. 5C-a). However, interestingly the slope of the opening rate changes was steeper in a voltage range where inward currents were elicited (−Vp = −80 to −40 mV: the closed column in Fig. 5C-b) than in a voltage range where outward currents were elicited (−Vp = +40 to +120 mV: the open column in Fig. 5C-b). By contrast, as the membrane voltage increased, the closing rate decreased under conditions with outward currents (−Vp = +40 to +120 mV: Fig. 5D-a and the open column in Fig. 5D-b), whereas it was unchanged under conditions with inward currents (−Vp = −80 to −40 mV: Fig. 5D-a and the closed column in Fig. 5D-b). Thus, the slopes of both the opening and closing rates abruptly changed with the reversal potential as the boundary, indicating the current-direction-dependent single-channel kinetics of mPanx1 channels.

**Figure 6:**
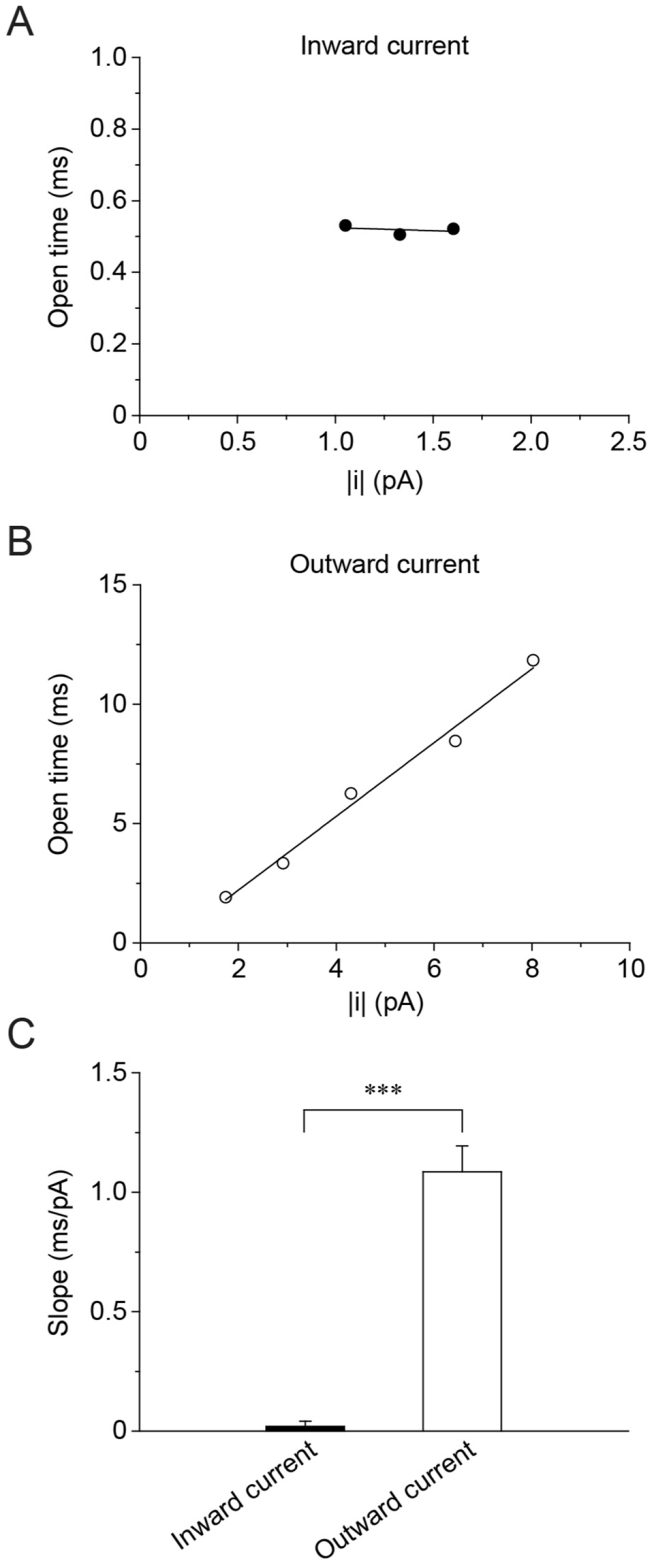
Direction and amplitude of single channel current control mPanx1 channel open time. Correlation between open time and |i| of mPanx1 channel with pipette and bath solutions containing (in mM) 140 NaCl, 2 KCl, 1 CaCl_2_, 1 MgCl_2_ and 10 HEPES. “|i|” indicates absolute value of single-channel current. No significant correlation was seen in hyperpolarization (Cl^−^ efflux; inward current) (r = 0.13) (**A**), while a strong positive correlation can be observed in depolarization (Cl^−^ influx; outward current) (r = 0.99) (**B**). The slope value in depolarization (1.09 ± 0.11 ms/pA, n = 5) was significantly larger than that in hyperpolarization (0.02 ± 0.02 ms/pA, n = 5) (**C**). Asterisk indicates significantly difference between inward and outward currents (***p < 0.001). Data are shown as mean ± SEM (n = 5).

Figure 6A and B show the correlation between open time and “|i|”; the absolute value of the single channel current. No significant correlation was seen in Cl^−^ efflux (inward current; hyperpolarized) conditions (r = 0.13) (Fig. 6A), whereas a strong positive correlation was observed in Cl^−^ influx (outward current; depolarized) conditions (r = 0.99) (Fig. 6B). The slope under Cl^−^ influx (outward current; depolarized) conditions (1.09 ± 0.11 ms/pA, n = 5) was significantly higher than that under Cl^−^ efflux (inward current; hyperpolarized) conditions (0.02 ± 0.02 ms/pA, n = 5) (Fig. 6C). These results suggest that the mean open time (closing rate) depends on the magnitude of charge carrier flux through the channel (|i|) under Cl^−^ influx (outward current; depolarized) conditions, and that ‘the direction of ionic flux’ through the channel plays a key role in the regulation of channel gating: *i.e*., the single channel closing kinetics of mPanx1 channel shows current-direction/amplitude-dependency.

### The gating of mPanx1 channel is regulated by the amplitude of unitary outward currents

**Figure 7:**
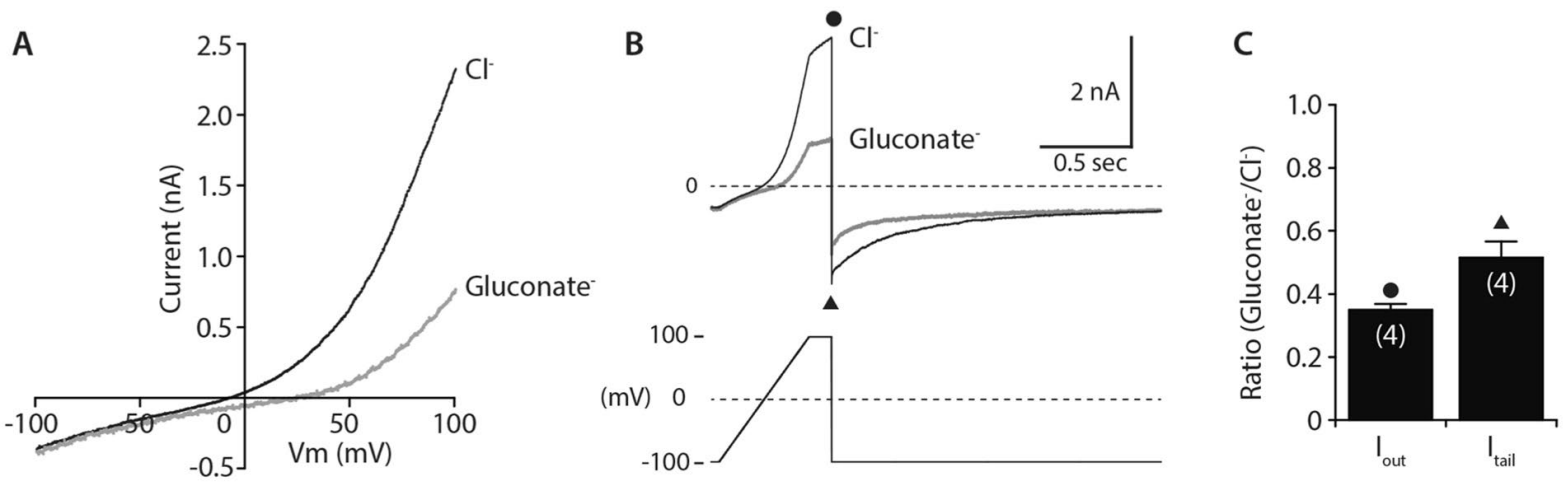
Current direction- and amplitude-dependent gating of mPanx1 is independent of membrane potential. (**A**) Neuro2a cells transiently transfected with mPanx1Δ371 were held at −100 mV and currents were recorded using a voltage ramp pulse (−100 to +100 mV, 1 s duration). The intracellular solution contained (in mM) 140 CsCl, 5 NaCl, 5 MgCl_2_, 5 EGTA and 10 HEPES, pH 7.4, and the NaCl bath solution contained (in mM) 140 NaCl, 5 KCl, 2 CaCl_2_, 1 MgCl_2_, 10 glucose and 10 HEPES, pH 7. To substitute Cl^−^ with gluconate^−^ in the bath, NaCl was replaced with equimolar Na-gluconate. Replacement of extracellular Cl^−^ with gluconate^−^ reduced the outward current amplitude and shifted E_rev_ to more positive potentials. (**B**) External Cl^−^ to gluconate^−^ replacement decreased not only the outward currents at +100 mV (I_out_, ⦁) but also the tail currents at −100 mV (I_tail_, ▴). (**C**) Ratios of I_out_ and I_tail_ in Na-gluconate and NaCl bath solutions are plotted. Data are shown as mean ± SEM (n = 4).

In the single channel analyses of cell-attached patch clamp recordings, we found that the mean open time of mPanx1 channels under Cl^−^ influx (outward current; depolarized) conditions appears to be linearly correlated with the amplitude of unitary outward currents (|i_out_|) (Fig. 6B). However, a correlation also exists between the mean open time and the membrane potential, because different |i_out_| were achieved by changing the membrane potential. To determine which parameter, |i_out_| or membrane potential, is actually correlated with the mean open time of mPanx1, we employed tail current analysis using the whole-cell patch clamp configuration. HEK293 and Neuro2a cells were transiently transfected with wild-type mPanx1 and membrane currents were recorded during voltage ramps from −100 to +100 mV from a holding potential of −100 mV. Consistent with the previous findings^25^, expression of mPanx1 generated currents in both cell lines (427.4 ± 118.2 and 391.5 ± 75.0 pA at +100 mV in HEK293 and Neuro2a cells, n = 9 and 5, respectively), which were too small for analysis of tail currents. We therefore created a mutant of mPanx1 (mPanx1Δ371) that lacks the C-terminal auto-inhibitory region (residues 371–426)^25^. Expression of mPanx1Δ371 generated large enough outwardly-rectifying currents (2037 ± 330 pA at +100 mV, n = 4, in Fig. 7A and B) for tail current analysis: clearly identifiable tail currents at −100 mV (529.0 ± 111.9 pA, ▴ in Fig. 7B) in Neuro2a cells. Outward currents at +100 mV (I_out_) were taken at the end of the pulse (⦁) and the tail currents (I_tail_) were calculated by subtracting the holding currents at −100 mV from the peak inward currents observed immediately after repolarizing from +100 mV to −100 mV (▴). This suggests that mPanx1Δ371 channels retain the P_o_ dependence of either |i_out_| or membrane potential.

In the voltage pulse protocol used here, the outward current at +100 mV (I_out_) reflects the rate of influx of the major extracellular anion, but the tail current (I_tail_) is carried by Cl^−^ efflux from the intracellular space and should not depend upon extracellular anions. Its amplitude is linearly related to the product of: 1) the P_o_ of the channel at the end of the depolarization pulse (+100 mV), and 2) the driving force of the Cl^−^ efflux at −100 mV, which is determined by the reversal potential (E_rev_). As expected, replacement of extracellular Cl^−^ with gluconate^−^ diminished |i_out_|, but, surprisingly, reversibly reduced the amplitude of I_tail_ as well as I_out_ (Fig. 7A–C). Because of the lower permeability of gluconate^−^ to the channel than Cl^−^ (Fig. 3), this anion substitution causes a positive shift in the reversal potential (E_rev_) as observed in Fig. 7A, thereby increasing the driving force for I_tail_ by 37% (considering approximate relative permeability of Cl^−^: gluconate^−^ = 1.0: 0.186 (Fig. 3C), the calculated E_rev_ in extracellular Cl^−^ or gluconate^−^ is 1.1 or 38.5 mV, respectively). Nevertheless, I_tail_ recorded in extracellular gluconate^−^ was 48.4% smaller than that in extracellular Cl^−^ (Fig. 7C), indicating that the P_o_ of the channel at +100 mV is 62.3% lower in gluconate^−^ than in Cl^−^. These data clearly demonstrate that the P_o_ of mPanx1 is regulated by the magnitude of |i_out_| independently of the membrane potential, strongly supporting the concept of the current-dependent gating of mPanx1.

## Discussion

In the present study, we investigated the gating properties of mPanx1 channels expressed in HEK293 and Neuro2a cells at the single-channel level by the cell-attached and whole patch-clamp techniques and found that their kinetics are drastically changed by voltage stimuli around the reversal potential (E_rev_). This voltage-dependence of the kinetics of mPanx1 channels may be due to electrostatic interactions between the surface potential and extracellular loops and/or the cytoplasmic N- and C-termini that have many charged residues and may interact with the gate in a voltage-dependent manner. Although the channel gating kinetics appears to depend on voltage (membrane potential), our results indicate that they are instead regulated in a manner that depends upon the direction and amplitude of currents through the channel. This result suggests that channel gating is not regulated directly by voltage, but is rather directly regulated by the direction and magnitude of charge carrier flux (current) through the channel. Because currents through the channel can occur in either direction, mPanx1 channels demonstrate apparent voltage-dependence.

**Figure 8:**
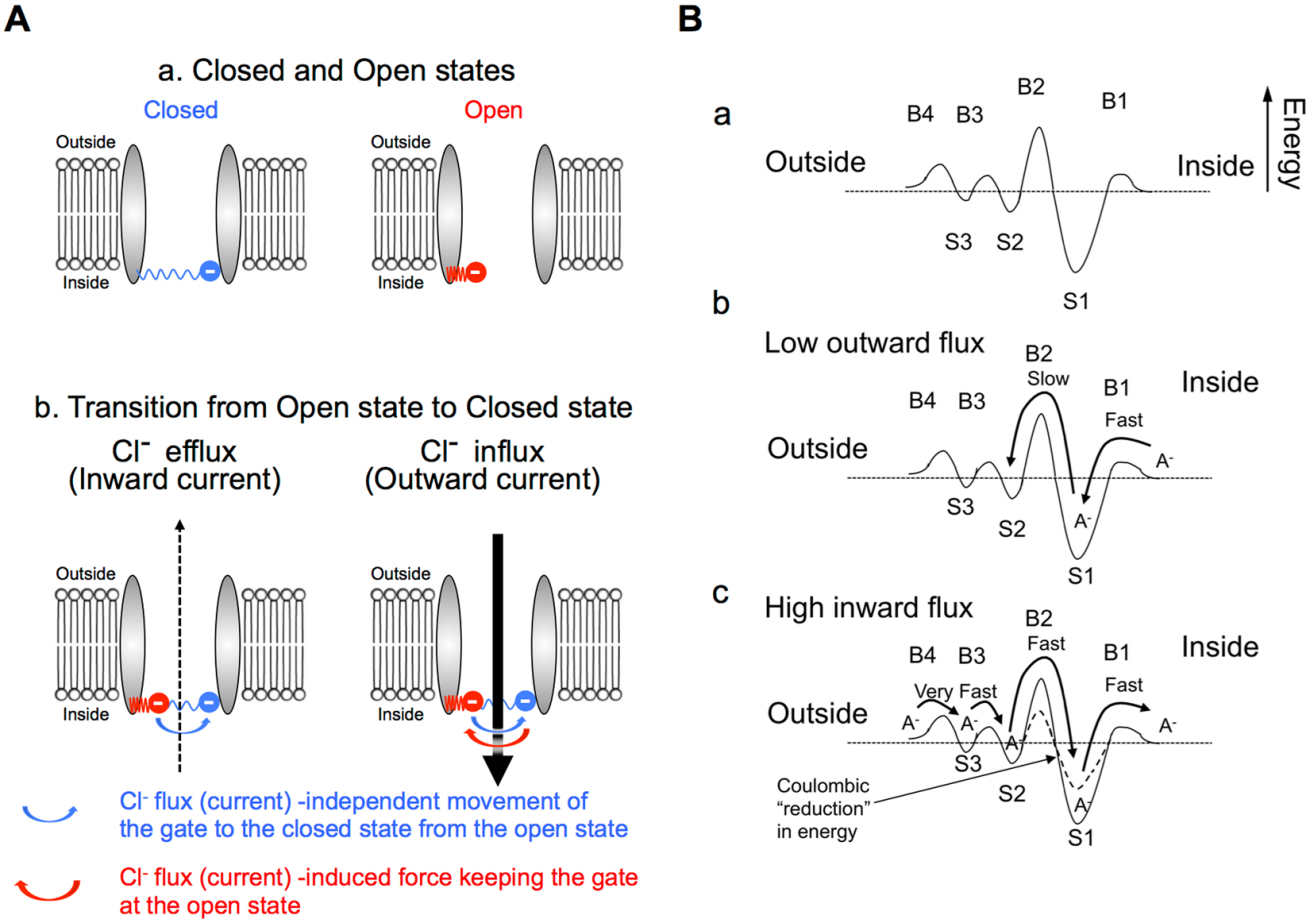
Ion-flux-dependent models of mPanx1 channel kinetics. (**A**) A schematic model of closed and open states. In this model, we assume a ‘negatively’ charged gate at the ‘cytoplasmic region’ in the channel pore. (a) The channel gate located on the right side in the channel pore (a blue circle with –) shows the closed state (left panel), while the channel gate located on the left side in the channel pore (a red circle with –) shows the open state (right panel). (b) Transition of the channel from the open state to the closed state. Under Cl^−^ efflux (inward current: hyperpolarized) conditions (left panel), the channel gate moves spontaneously toward the closed location from the opened location as indicated by a blue arrow without any Cl^−^-efflux (inward-current) -induced force affecting the channel gate movement. In contrast, under Cl^−^ influx (outward current: depolarized) conditions (right panel), the force (a red arrow) induced by the Cl^−^ influx (outward current) keeps the channel gate open in a manner dependent on the magnitude of the Cl^−^ efflux (outward current) against the spontaneous closing movement of the channel gate (a blue arrow). (**B**) A hypothetical energetic model of ion flux (current) through the mPanx1 channel. (a) The mPanx1 channel is represented as a series of multiple anionic binding sites S1 – S3 and multiple energetic barriers to ion movement, B1 – B4. Barrier B2 is relatively high and binding site S1 is relatively deep. (b) The situation for outward Cl^−^ movement (inward current). Cl^−^ enters the channel from the cytosol inside the cell and binds to the strong binding site, S1. After binding, Cl^−^ will usually return to the cytosol but occasionally will acquire enough energy to cross the adjacent barrier B2 and continue across the multiple small barriers and weak binding sites to the exterior of the cell. Crossing barrier B2 would be associated with channel gating. Thus, since the rate limiting step is crossing barrier B2, the Cl^−^ efflux (inward current) would be small, and the gate is quickly closed. (c) The situation for anion influx (outward current). Cl^−^ enters the outer mouth of the channel easily and multiple ions (Cl^−^) rapidly occupy all of the weak binding sites, S3 and S2. The buildup of multiple anionic (Cl^−^) charges in the outer part of the channel provides additional coulombic energy to reduce energy barrier B2 (dotted line: the difference between the levels of barrier B2 and binding site S2 reduces to the dotted line from the solid line due to the buildup of S2 level by multiple anionic (Cl^−^) charges) and allow ion (Cl^−^) movement to the strong binding site S1 near the cytosolic surface, but coulombic force also provides energy to move anions (Cl^−^) out of this well into the cytosol (dotted line). Thus, Cl^−^ influx (outward current) is large, and the gate is kept continuously open depending on the Cl^−^ influx (outward current).

A model of the channel gating is shown in Fig. 8A, in which we assume that the gate contains a charged residue(s) and is located within the pore but not in the transmembrane region (*i.e*., in extracellular or intracellular portion of the pore). As shown in Fig. 8A-a, if we assume a ‘negatively’ charged gate at the ‘cytoplasmic region’ in the pore, the channel gate located on the right side in the channel pore (a blue circle with – in Fig. 8A-a, left panel) shows the closed state, while the channel gate located on the left side in the channel pore (a red circle with – in Fig. 8A-a, right panel) shows the open state. Figure 8A-b indicates the transition of the channel from the open state to the closed state. Under Cl^−^ efflux (inward current: hyperpolarized) conditions (Fig. 8A-b, left panel), the channel gate closes spontaneously (the channel gate moves from the red circle location (open sate) to the blue circle location (closed state) as indicated with a blue arrow in Fig. 8A-b, left panel) without any Cl^−^ efflux (inward) -current-induced force affecting the channel gating movement. In contrast, under Cl^−^ influx (outward current: depolarized) conditions (Fig. 8A-b, right panel), the force (a red arrow in Fig. 8A-b, right panel) induced by the Cl^−^ influx (outward current) keeps the channel gate open in a manner dependent on the magnitude of the Cl^−^ influx (outward current) against the spontaneous movement of the channel gate to the closed state (a blue arrow in Fig. 8A-b, right panel). Thus, the length of the channel open time increases as the amplitude of single channel outward current increases. The closed time (opening rate) may be controlled by interactions between “gating charges” and membrane potential.

The behavior of Panx1 is reminiscent of certain types of K^+^ channels, in which the magnitude of current and the rate of gating depend upon the direction of ion flux^45–49^. All of these channels have multiple binding sites and multiple asymmetric energy barriers to ion flux. The channel is modeled by a chain of saturable binding sites that can be occupied by a number of ions. The ions move in single file to one of its immediate neighboring vacant sites with absolute rates determined through Eyring equation by the barrier heights between sites, the membrane potential, and the ion-ion repulsion. The Panx1 channel appears to follow this paradigm. For the Panx1 channel, we can approximate the properties of the channel if the pore of the channel has a strong binding site (deep energy well) near the cytosolic surface with an adjacent high energy barrier to ion flux. In addition, the channel would need to have multiple (at least 2) weak binding sites and small barriers near the extracellular end of the channel pore (see Fig. 8B in detail).

The voltage-sensing mechanisms of voltage-gated channels are of general interest. In many channels, voltage gating is regulated by a voltage sensor domain within the transmembrane domain, for example the S4 domain of the super-family of voltage-gated K^+^, Na^+^ and Ca^2+^ channels^50, 51^. However, Panx1 lacks an obvious voltage sensor domain. Other ion channels and receptors are voltage-dependent despite the absence of a voltage sensor. For example, nicotinic ACh receptors, P2X_2_ receptors^52, 53^ and some metabotropic glutamate receptors, including mGluR1a and mGluR3^54^, have ligand binding sites at either the extracellular or intracellular sides with ligand binding causing conformational changes that produce an apparent voltage dependence^52–54^. Replacement of extracellular Cl^−^ with other anions, including MeSO_3_^−^, Br^−^, I^−^ and SCN^−^, modulates the gating properties of ASIC1a recorded in rat hippocampal neurons with the whole-cell patch-clamp technique^27^. ASIC1a channels consist of three subunits with a trimeric extracellular domain containing three “anion-binding sites” that are highly conserved among ASIC isoforms^55^. Cl^−^ regulates ENaC gating^36, 56^.

In contrast, our results here demonstrate that mPanx1 channels show apparent voltage-dependent gating kinetics (Fig. 4A), but the voltage dependence is not mediated directly by membrane potential but rather by the direction and amplitude of currents (anion flux). Thus, mPanx1 may not require a voltage sensor to gate. However, mPanx1 has charged residues located at the cytoplasmic side of the channel. It is plausible that some of these charged residues are located within the electric field across the plasma membrane and act as voltage sensors, causing conformational changes that lead to modulation of channel gating. Alternatively, the present study supports a novel model in which the direction and amplitude of currents through the channel influence channel gating, providing voltage dependence mediated by interactions of ions in the permeation with the channel gating machinery.

In summary, the present study suggests that the direction and magnitude of charge flow regulate the ‘voltage-dependent’ single-channel gating kinetics of mPanx1 channel, thus providing a novel model in which single channel gating kinetics are dependent upon the direction and amplitude of currents through the channel.
